# Historical land-use and landscape change in southern Sweden and implications for present and future biodiversity

**DOI:** 10.1002/ece3.1198

**Published:** 2014-09-01

**Authors:** Qiao-Yu Cui, Marie-José Gaillard, Geoffrey Lemdahl, Li Stenberg, Shinya Sugita, Ganna Zernova

**Affiliations:** 1 Department of Biology and Environmental Science, Linnaeus UniversityKalmar, SE-39182, Sweden; 2 Department of Earth- and Ecosystem Sciences, Lund UniversityLund, SE-223 62, Sweden; 3 Institute of Ecology, University of TallinnTallinn, 10120, Estonia

**Keywords:** Biodiversity, fossil pollen records, historical maps, Landscape Reconstruction Algorithm, land-use and landscape changes, late Holocene, southern Sweden

## Abstract

The two major aims of this study are (1) To test the performance of the Landscape Reconstruction Algorithm (LRA) to quantify past landscape changes using historical maps and related written sources, and (2) to use the LRA and map reconstructions for a better understanding of the origin of landscape diversity and the recent loss of species diversity. Southern Sweden, hemiboreal vegetation zone. The LRA was applied on pollen records from three small bogs for four time windows between AD 1700 and 2010. The LRA estimates of % cover for woodland/forest, grassland, wetland, and cultivated land were compared with those extracted from historical maps within 3-km radius around each bog. Map-extracted land-use categories and pollen-based LRA estimates (in % cover) of the same land-use categories show a reasonable agreement in several cases; when they do not agree, the assumptions used in the data (maps)-model (LRA) comparison are a better explanation of the discrepancies between the two than possible biases of the LRA modeling approach. Both the LRA reconstructions and the historical maps reveal between-site differences in landscape characteristics through time, but they demonstrate comparable, profound transformations of the regional and local landscapes over time and space due to the agrarian reforms in southern Sweden during the 18th and 19th centuries. The LRA was found to be the most reasonable approach so far to reconstruct quantitatively past landscape changes from fossil pollen data. The existing landscape diversity in the region at the beginning of the 18th century had its origin in the long-term regional and local vegetation and land-use history over millennia. Agrarian reforms since the 18th century resulted in a dramatic loss of landscape diversity and evenness in both time and space over the last two centuries leading to a similarly dramatic loss of species (e.g., beetles).

## Introduction

Reconstruction of past vegetation/landscape change in quantitative terms (e.g., percentage (%) cover or biomass of plant taxa) at both regional and local spatial scales may be required to answer questions related to past land cover-climate interactions (e.g. Gaillard et al. 2010; Strandberg et al. 2014), landscape management and biodiversity conservation (e.g., Smith et al. 2010; Lindbladh et al. 2013), human impact on natural vegetation (e.g., Nielsen and Odgaard 2010; Fyfe et al. 2013), and general land cover/land use – environment relationships (e.g., Dearing 2006). Pollen-based reconstruction of past vegetation abundance in quantitative terms has long been a challenge for palynologists (Sugita 1994). This is due to the fact that pollen loading in lakes or bogs includes two components: (1) the local pollen coming from plants within a relevant source area of pollen (RSAP sensu Sugita 1994; see definition below) and (2) the background pollen coming from plants outside the RSAP. The relative proportion of these two components changes in space and time and depends on basin size and the plant taxa involved (Sugita 1994). Moreover, intertaxonomic differences in pollen productivity and pollen dispersal and deposition produce biases that prevent the use of both PARs and pollen percentages as direct proxies of plant abundance (e.g., Andersen 1970; Prentice 1985; Sugita 1994; Cui et al. 2013).

The Landscape Reconstruction Algorithm (LRA, Sugita 2007a,b) overcomes those problems by calculating the relevant source area of pollen (RSAP, sensu Sugita 1994) and the background pollen component for a specific small-size site and its pollen record(s). The RSAP is the distance from the center of a lake/bog for which the pollen–vegetation relationship does not improve, and the background pollen remains the same within a given landscape (Sugita 1994). It is also the smallest spatial unit for which vegetation abundance can be estimated by the model using fossil pollen records (Sugita 2007b). The LRA includes two models: Regional Estimates of Vegetation Abundance from Large Sites (REVEALS) and Local Vegetation Estimates (LOVE). The REVEALS model was tested theoretically using a simulation approach (Sugita 2007a) and empirical modern and historical vegetation data in NW Europe and North America (Hellman et al. 2008a,b; Nielsen and Odgaard 2010; Soepboer et al. 2010; Sugita et al. 2010). The LRA approach (REVEALS + LOVE) was tested in northern America (Sugita et al. 2010) (pollen records from small forest hollows), in Denmark (Nielsen and Odgaard 2010; Overballe-Petersen et al. 2013), and in southern Sweden (Fredh 2012; Mazier et al. in press). These tests suggest that the REVEALS model and LRA approach are robust methods to infer regional and local vegetation cover using pollen records from large and small sites, respectively.

**Figure 1:**
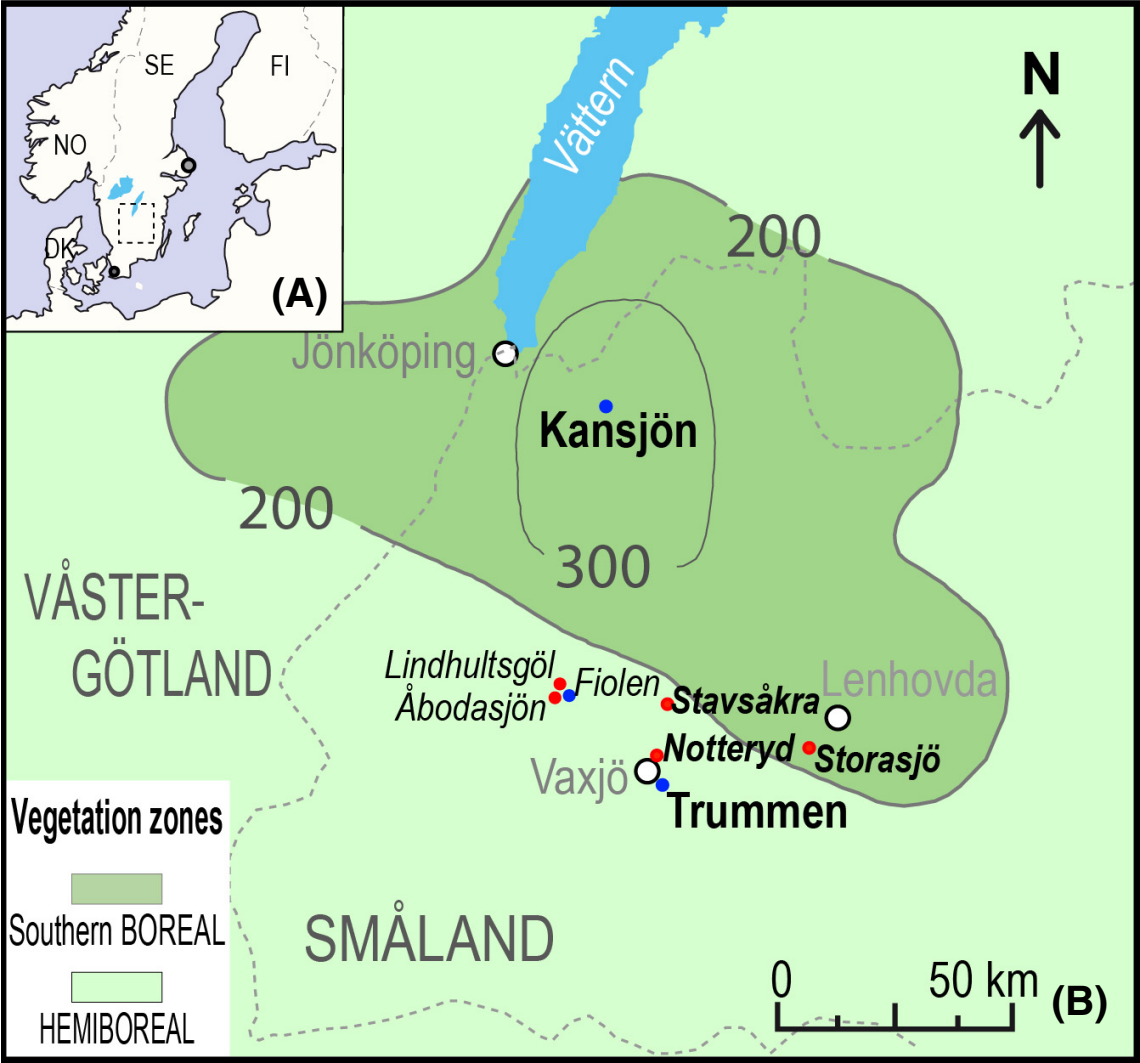
Study area. (A) Location of the investigation area in southern Sweden. B: location of the sites with pollen records used in the Landscape Reconstruction Algorithm (red dots: bogs; blue dots: lakes). The 200 m a.s.l. isoline delimits the Småland Uplands and corresponds approximately to an enclave of south-boreal vegetation within the hemiboreal vegetation zone of southern Sweden (Ahti et al. 1968). Dashed lines: limits of the province of Småland. Abbreviations: DK, Denmark; FI, Finland; NO, Norway; SE, Sweden.

The hemiboreal vegetation zone of southern Sweden (Ahti et al. 1968; Fig. 1) is particularly interesting to study biodiversity issues in NW Europe. The hemiboreal zone’s southern boundary corresponds to the southern natural distribution limit of *Picea abies* (spruce), and its northern boundary largely follows the northern distribution limit of *Quercus robur* (pedunculate oak). The province of Småland (Fig. 1) covers a large part of the Swedish hemiboreal zone. Moreover, the region named “Småland Uplands” is an enclave of southern boreal vegetation within the hemiboreal zone (Fig. 1). Although the hemiboreal zone is more closely related to the boreal than the temperate vegetation zone, it has elements from both the coniferous forests of the boreal zone and the true deciduous forests of the temperate zone. This explains that it is characterized by a particularly high number of species and habitats (e.g., Gustafsson and Ahlén 1996).

Today, the hemiboreal and southern boreal zones of southern Sweden are characterized by modern forestry with plantations of spruce and pine. However, some areas are still characterized by high species and habitat diversity, and the conditions required for its maintenance still are much debated by ecologists and paleoecologists (e.g., Björkman, 1996; Hannon et al. 2010; Lindbladh et al. 2003, 2007, 2008; Niklasson et al. 2002). Various factors have been put forward as important for long-term biodiversity; for instance, long continuity of natural woodland vegetation and fire disturbances have been suggested as past conditions required for the maintenance of rare beetle species (e.g., Niklasson et al. 2002; Hultberg et al. 2014). The importance of long-term, regional-scale fire disturbances was recently demonstrated in a study of two sites in the province of Småland (Olsson and Lemdahl 2009, 2010; Cui et al. 2013). Hultberg et al. (2014) also propose that, besides local, long-term continuity of woodland and fire disturbances, ancient agrarian activities such as woodland grazing and hay making probably did favor high species and habitat diversity, and therefore high beetle diversity. In contrast, the importance of the regional landscape structure in terms of number and distribution of habitat and vegetation types has not been much discussed so far (see, however, Olsson and Lemdahl 2009, 2010; Cui et al. 2013). This is mainly due to the fact that individual paleoecological investigations provide information on the local environmental history a few kilometers around the study sites and do not inform on the distribution of vegetation types in the landscape. Therefore, the combined interpretation of pollen-based reconstructions of past landscapes and historical sources (maps and associated written documents) from several sites in a region such as the province of Småland might offer, on the one hand, the necessary regional and spatial dimension that is missing in paleoecological data and, on the other hand, the plant taxonomical detail and long-term perspective that is missing in the historical data.

The major objectives of this study are (1) to reconstruct the major landscape transformations during the last three centuries in the study region using both historical sources and pollen-based vegetation and landscape reconstructions, and (2) to discuss the causes behind the main landscape changes in the past and their implications for biodiversity issues. In order to achieve this goal, we use pollen records from three small bog sites and two large lake sites in the province of Småland (Fig. 1) and related historical documents. We first test the Landscape Reconstruction Algorithm (LRA) by comparing the cover of various vegetation/land-use types obtained from (1) pollen-based LRA estimates and (2) a series of historical maps from AD 1685 to AD 1210. We then evaluate the pros and cons of pollen records and historical documents for reconstruction of historical land-use and landscape changes. Finally, based on the combined pollen-based LRA reconstructions and the historical data, we draw conclusions on the possible implications of the landscape transformations of the last three centuries for the present and future biodiversity of the region.

## Material and Methods

Hereafter, the taxonomy and nomenclature of plant taxa and pollen taxa follow Flora Europaea (Tutin et al. 1964) and the European Pollen Database (Giesecke et al. 2013; http://www.europeanpollendatabase.net/about), respectively. The plant names are given in both Latin and English the first time, then in either Latin or English. The term “forest” is used for areas planted with trees and managed as part of a modern forestry system (from the 20th century), while the term “woodland” refers to natural tree vegetation that may be used by humans in an old-fashioned, traditional way, for example grazing, wood collection, small-scale hay making, and slash and burn cultivation.

### Study sites and land-use history

The application of the Landscape Reconstruction Algorithm (LRA) (including the two models REVEALS and LOVE) (Sugita 2007a,b; see section on the LRA below) requires pollen records from large and small sites. We are using the records from (1) the two large sites Trummen and Kansjön for application of the REVEALS model and (2) the three small sites Stavsåkra, Notteryd, and Storasjö for the application of the LOVE model (Fig. 1; see Appendix S1 for further descriptions of the sites). The land-use history in the study region was strongly influenced by three major agricultural reforms in southern Sweden from the 1780s:

1. the “Storskifte” land reform or the “one land per farm” reform (AD 1772–1825 in Småland), the main purpose of which was to redistribute the land so that each farm had one large piece of land instead of many scattered small pieces, which was thought to be negative for efficiency and high production.
2. the “Enskifte” land reform (ca. AD 1825–1922 in Småland) urged landowners to move their farms out of the village and get their land consolidated into one piece and enclosed.
3. the “Lagaskifte” land reform (ca. AD 1827–1922 in Småland) allowed more than one piece of land per landowner so that each farm got its share of the “outfields” (or commons, i.e. forest and pasture land used in common by a village in the old “infield/outfields” system existing before the reforms), one piece of meadowland, and one piece of arable land (both being part of the “infield” area of a village) (Helmfrid 1961).

### Test of the Landscape Reconstruction Algorithm: model-data comparison

The performance of the Landscape Reconstruction Algorithm (LRA) can be evaluated by comparing the pollen-based LRA reconstructions of vegetation cover with values of vegetation cover extracted from modern and historical data sets such as modern vegetation inventories and economical maps, and historical maps with their related documents. The LRA reconstructions provide estimates of the percentage cover of the major taxa occurring in the pollen records (in this study, 25 taxa; see below for more details), while modern and historical maps provide the percentage cover of major land-use/landscape/vegetation units (LuVs). Therefore, there is a mismatch between the LRA reconstructions (% cover of individual taxa) and the vegetation data from maps (% cover of LuVs). Although some modern vegetation maps might include information on the percentage cover of individual taxa within each vegetation unit, it is not always the case. Moreover, pollen records are seldom from sites located in areas for which detailed vegetation maps are available. Historical maps do not provide details on the taxa composition within each LuV. Therefore, if LRA reconstructions and map data are to be compared, it implies a “transformation” of the original data of either LRA % cover of taxa or LuV % cover extracted from maps. Two approaches may be envisaged: (1) the LRA % cover of individual taxa are summed up into % cover of LuVs by assuming the taxa composition of each LuV, and (2) the LuVs % cover from maps are translated into % cover of individual taxa by assuming the % cover of taxa within each LuV. In both cases, assumptions on the taxa composition of LuVs can be more or less complex. The simplest assumption implies that each taxon occurs in only one LuV. If each taxon is assigned to several LuVs, the assumptions on the taxa composition of each LuV will be more complex, because the % cover of one taxon will have to be assumed for several LuVs.

The more complex the assumptions are, the more difficult the evaluation of discrepancies between the LRA model’s results and the maps’ data will be. Therefore, the simplest assumptions should be favored. So far, only two tests of the LRA’s performance were performed in Europe, in eastern Denmark (Overballe-Petersen et al. 2013), and in southern Sweden (Fredh 2012; Mazier et al. in press; Fig. 1). In both tests, the LRA estimates (% cover) of individual taxa (LRA-taxa) were grouped into LRA estimates of maps’ LuVs (LRA-LuVs). However, Overballe-Petersen et al. (2013) grouped the LRA-taxa into LRA-LuVs only very broadly similar to the maps’ LuVs. Moreover, harmonization of the LuVs between different types of historical maps was not attempted. Mazier et al. (in press) grouped the LRA-taxa into LRA-LuVs corresponding as closely as possible to the maps’ LuVs; however, harmonization of the LuVs between the different types of maps was not performed between the oldest maps and the more recent ones. Therefore, the LRA-taxa were grouped into different LRA-LuVs depending on the map type.

In this study, we aimed at being as consequent as possible in our approach, in order to facilitate the evaluation of the model-data comparison and the identification of possible causes behind discrepancies between LRA reconstructions (LRA-LuVs) and map data (map-LuVs). To achieve this, we (1) harmonized the LuVs between different types of maps using the descriptions of the land-use types in the historical documents, (2) chose the simplest assumption described above, that is assigned each pollen taxon to a single LuV using the same information as under (1) above, and general knowledge on the current and historical vegetation of southern Sweden, and (3) assumed an even composition of taxa within each LuV, that is a LuV characterized by two or four taxa was assumed to have a cover of 50%, respectively, 25% of each taxon. Moreover, for taxa known to be characteristic of several LuVs such as *Calluna* (heather), we used the multiscenario approach in order to identify more easily the possible causes behind discrepancies between LRA estimates of LuVs and map-extracted LuVs. For instance, for comparison with the map-LuVs, we used three alternatives of LRA-LuVs where *Calluna* was included in either (1) LuV woodland, (2) LuV wetland, or (3) LuV grassland (see below for more details).

### Modern and historical land-use/vegetation data

#### The maps

**Figure 2:**
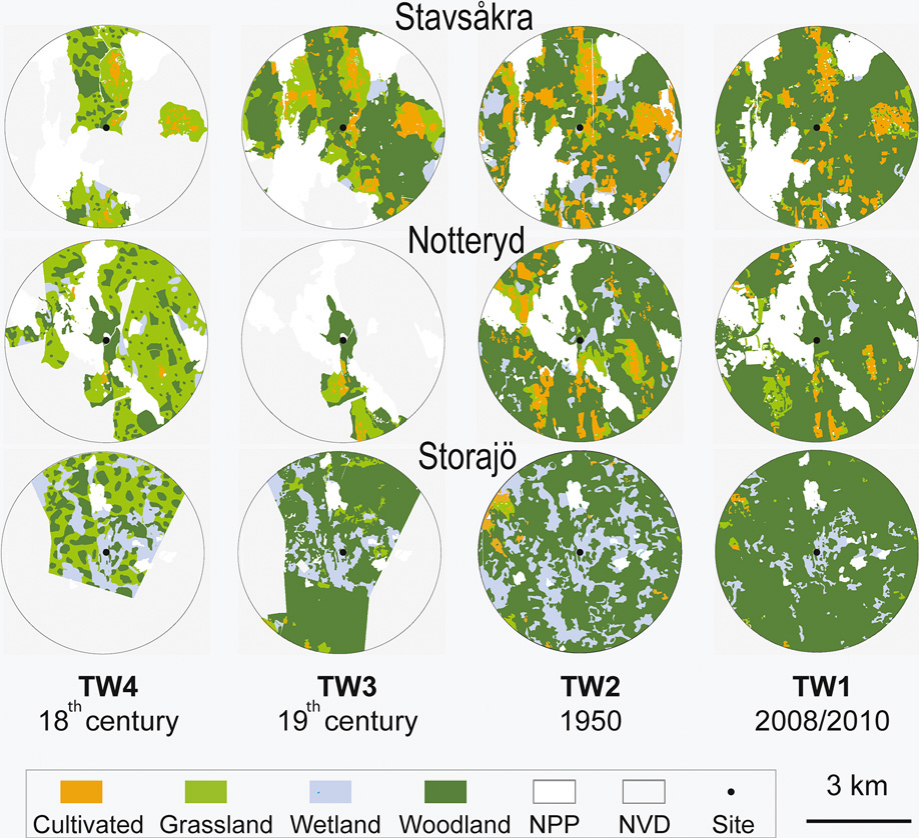
Harmonized land-use/vegetation (LuV) categories within 3-km-radius circles around the three small pollen sites Stavsåkra, Notteryd, and Storasjö (dark dot) interpreted from historical and modern vegetation maps (see Table 2 for details) for four time windows: AD 1950–2010 (TW1), AD 1925–1950 (TW2), AD 1825–1925 (TW3), and AD 1700–1825 (TW4). Abbreviations: LuV1, Cultivated land; LuV2, Grassland; LuV3, Wetland; LuV4, Woodland/Forest; NPP, Non-pollen producing areas; NVD, no historical map data.

A large number of cadastral maps (i.e., belonging to a “cadaster”) documenting the reforms described above were produced in different years depending on the village or the commune. These maps show the boundaries and ownership of land parcels such as woodlands or forests, meadows, and cultivated land. The cadaster also includes documents (often related to the maps) that provide details of the ownership, the dimensions (area), the cultivations if rural, and the value of individual parcels of land. All available modern and historical cadastral maps within a 3-km-radius circle around each of the three study sites were collected from the Swedish Land Survey and grouped into four time windows (TW) based on the time of the major land reforms (Table 1). Each original map was scanned. ArcGIS was used for rectification, digitization, visualization, and extraction of the vegetation information. The final historical maps used for extraction of land-use/vegetation data are digitized joint maps, one for each of the four TWs 1–4 at the two study sites (Fig. 2) (see also Appendix S1 for further details on the maps and methods used). Each joint map does not necessarily cover the entire surface of the 3-km-radius circle around each site, especially for the oldest TWs. We assume that the areas not covered by the joint maps are characterized by the same vegetation as the areas covered by the maps.

**Table 1:** Information on the pollen data used in the Landscape Reconstruction Algorithm (LRA) application (age of stratigraphical levels with pollen data and total pollen count) and the time periods covered by the historical maps used for testing the LRA

		Pollen counts used in LRA			
		REVEALS (K/T)		LOVE (STAV/NOTT/STOR)	
Time Windows (TW)	Maps (STAV, NOTT STOR)	Age	Total pollen count	Age	Total pollen count
TW1 AD 1950–2010	Topographic map (2008, 2010)	1950/1950	2536	1930/1993/1935	617/841/574
TW2 AD 1925–1950	Economy map (1950)				
TW3 AD 1825–1925	Lagaskifte Period (1825–1922)	1780/1745	2557	1870/1900/1895	264/568/504
TW4 AD 1700–1825	Storskifte Period (1772–1825)			1690–1790 (4 samples)/1650–1785(3 samples)/1723,1815	2017/3067/962
	Older maps^1^ (1700–1740)				

K, Kansjön; T, Trummen; STAV, Stavsåkra; NOTT, Notteryd; STOR, Storasjö.

The age is in calibrated year (AD).

1 Older maps only available at site Stavsåkra.

#### Harmonization of land-use/vegetation classes

Because the definition of the original land-use/vegetation classes (LuVs) differs between historical maps within and between time windows, we chose to harmonize those definitions and simplified the classification into four harmonized map-LuVs. In the following text, LuVs 1–4 refer to the four harmonized classes: (1) cultivated land, (2) grassland, (3) wetland, and (4) woodland/forest. The harmonization of LuVs (Table 2; Fig. 2) implied assumptions based on expert knowledge of local vegetation and land-use history in the study area (see Appendix S1).

**Table 2:** Harmonization of the land-use/vegetation (LuV) classes for Landscape Reconstruction Algorithm (LRA) estimates and historical maps. **LRA-based LuV (%) is the sum of LRA estimates (%) of all taxa assigned to the LuV; Map-based LuV (%) is the sum of distance-weighted LuV (%) calculated from the maps’ LuV’s area (m^2^) divided by the total surface of the study area (in this case a 3-km-radius area around the pollen site. Three alternatives (^1,^ ^2,^ ^3^) of *Calluna* assignment to an LuV and two alternatives with exclusion of taxa (^4^*Calluna* and ^5^*Calluna* and Cyperaceae) are marked by superscript

Land-use/Vegetation (LuV) class used in this study	Map classes included in LuV classes	Pollen types included in the LRA estimates**
Cultivated Land (LuV1)	Cultivated land	*Cerealia*-t, *Scecal*e-t
Grassland (LuV2)	Meadow, Outland without treecover, Pasture, Mowed meadow on dry ground, Grassland, Open land	Comp. SF.Cich, *Filipendula*, *Juniperus*, *Plantago lanc.,* Poceae, *Potentilia*-t, *Ranuc. acrist*-t, Rubiaceae, *Rumex acetosa*-t, *Calluna vulg*.^(1)^
Wetland (LuV3)	Wetland, Bog, Fen, Open wetland, Mowed meadow on wet ground	Cyperaceae, *Salix*, *Calluna vulg*.^(2)^
Woodland/Forest (LuV4)	Forest, Outland, Wetland with tree cover, Deciduous forest	*Alnus*, *Betula*, *Carpinus*, *Corylus*, *Fagus*, *Fraxinus*, *Picea*, *Pinus*, *Quercus*, *Tilia*, *Ulmus*, *Calluna vulg*.^(3)^
Nonpollen producing (NPP)	Lake, Settlements, Deforestation/Clear cut	*Calluna vulg*.^(4)^; *Calluna vulg* & Cyperaceae^(5)^

t, type; *vulg*., *vulgaris*; *lanc*., *lanceolata*; Ranunc., *Ranunculus*; Comp. SF. Cich, Compositae SubFamily Cichorioideae.

#### Attribution of pollen types to the maps’ land-use/vegetation classes

In this study, we use 25 pollen taxa for which estimates of pollen productivity (PPEs) are available (see Appendix S1). We assume that each of the 25 plant taxa in the LRA reconstruction is characteristic of a single LuV and that the plant taxa within a LuV have an equal share of that LuV’s total cover (Table 2). These assumptions are not realistic, but they are consequent and imply that multiple assumptions on the share of taxa in each LuV are avoided. Instead, in order to handle the cases of taxa with possible distributions in more than one LuV, we used the multiple scenario approach. Because *Calluna* might have grown in woodlands, bogs, and/or grazed heaths (classified as “grassland”), and Cyperaceae (sedges) in wetlands, woodlands on wet soils, and/or grasslands, we examined five scenarios (referred below as *Calluna* scenarios I–V) of attribution of pollen taxa: *Calluna* in grassland (I), wetland (II) or woodland/forest (III), and *Calluna* excluded (IV) or *Calluna* + Cyperaceae excluded (V). The arguments for the attribution of taxa to LuVs are given in Appendix S1.

#### Distance-weighting vegetation abundance from historical maps

LRA reconstructions provide estimates of distance-weighted plant abundance for each LuV (see section on the LRA below). Therefore, the land-use/vegetation data extracted from the maps need to be distance-weighted (DW) for comparison with the LRA estimates (LRA-DWLuVs) (see Appendix S1 for details on the methods used to distance-weight vegetation data).

### Pollen data and LRA reconstruction of plant abundance

The LRA uses a two-step strategy to estimate distance-weighted abundance of plant taxa within the relevant source area of pollen (RSAP sensu Sugita 1994) of the investigated sites (lakes or bogs): 1) regional cover of plant taxa in an area of 10^4^–10^5^ km^2^ is estimated using pollen records from large sites (lakes or bogs ≥ 10^2^ ha) and the REVEALS model (Sugita 2007a), and 2) local cover of plant taxa within the RSAP is estimated using pollen records from small sites (lakes or bogs of a few hectares or smaller) and the LOVE model (Sugita 2007b). The LOVE model (step 2) estimates the RSAP and the mean distance-weighted plant abundance (DWPA) for each plant taxon within that RSAP using a backward modeling approach requiring REVEALS estimates of regional vegetation abundance (step 1 above) and pollen data from a number of small sites (Sugita 2007b; Sugita et al. 2010). The LOVE model is based on the ERV models of the pollen–vegetation relationship (Prentice and Parsons 1983; Prentice 1985) where pollen loading of a given plant taxon in a site (lake or bog) is linearly related to the taxon’s DWPA around that site multiplied by the taxon’s pollen productivity. Plant abundance is weighted so that plants close to the site have more weight (supply more pollen to the site) than those growing long from the coring site (supply less pollen to the site). More details on the LRA application are found in Appendix S1.

Below, “LRA reconstruction” and “LRA estimate” are synonymous with, respectively, “LOVE reconstruction” and “LOVE estimate,” a terminology used in other publications. The pollen-based LRA estimates of distance-weighted plant abundance (DWPA) and historical map-DWPA for LuV (1–4) are abbreviated LRA-LuVs, LRA grassland, LRA woodland/forest, etc., and map-LuVs, map-grassland, map-woodland/forest, etc., respectively. LRA estimates of DWPA for individual plant taxa are abbreviated LRA-taxon name (e.g., LRA-*Pinus)*.

## Results

### Historical maps

Below, we follow the definitions by Hellman et al. (2009a,b) for the concepts of “landscape diversity” and “landscape evenness.” “Landscape diversity” is high/low if the number of LuVs present at a small spatial scale of a few hundred square meters is high/low, and “landscape evenness” is high/low if the distribution and size of the LuV patches at the larger spatial scale (the 3-km-radius area around each site) are regular/irregular and small/large, respectively.

The harmonized historical maps for the four time windows (TWs; Fig. 2) show two major differences between the study sites: (1) the cover of wetland is much higher in the Storasjö area than in the Stavsåkra and Notteryd areas throughout the study period, and (2) the cover of grassland (TWs 3 and 4) and cultivated land (TW 1 and 2) is higher in Stavsåkra than in Notteryd and Storasjö. Changes in LuVs at Storasjö occur as two major and abrupt transformations: the first one between TW4 (18th century) and TW3 (19th century) as almost all grassland areas are replaced by woodlands/forests, and the second one between TW2 (AD 1950) and TW1 (AD 2008–2010) as most wetlands are replaced by woodlands/forests. These changes were accompanied by large decreases in landscape diversity and evenness, from a relatively even distribution of small patches woodland, grassland, and wetland in TW4 to a less even distribution of larger and smaller LuV patches with a strong dominance of woodlands and wetlands in TWs 3 and 2, and a very uneven and monotonous landscape in TW1 with an almost continuous cover of woodlands/forests with few small patches of wetlands and rare patches of grasslands and cultivated land. At Notteryd, the first major transformation occurs between sometime before TW2 (perhaps already between TW4 and TW3 as at Storasjö, although the historical maps are too few for TW3 to confirm it). At TW3, most grasslands occurring at TW4 are replaced by woodlands/forests in the eastern part of the area and by cultivated fields in the western and southern parts of the area. Between TW2 and TW1, most of the cultivated fields of the western part are replaced by woodlands/forests, grasslands, or wetlands, and several of the small patches of grassland, wetland, and cultivated land in the eastern part are replaced by woodlands/forests. Changes in landscape diversity and evenness are not as pronounced as in Storasjö, but there is a major change in landscape composition sometime between TW4 and TW2 from a dominance of grasslands to a dominance of woodlands/forests, which is reinforced between TW2 and TW1 and accompanied by a decrease in landscape evenness. At Stavsåkra, changes in landscape diversity and evenness are less pronounced through the study period than in Notteryd and Storasjö, but similar transformations are noticeable. The maximum landscape evenness is also found in TW4 as in Storasjö, with a relatively even distribution of small grassland and woodland patches and less abundant smaller patches of wetland and cultivated land. The decrease in grassland in favor of woodland and cultivated land between TW4 and TW3 implies a drop in landscape evenness as woodland forms larger patches than earlier, while grassland is more fragmented, and patches are smaller. In TW2, the landscape evenness is not very different from that in TW3, but cultivated land represents a larger portion of the landscape, and patches are larger than earlier, while grassland is even less common, more fragmented, and patches are very small. The landscape evenness decreases further between TW2 and TW1 as patches of grassland, wetland, and cultivated land become less numerous and smaller in size, and the cover of woodland/forest gets larger and more continuous than earlier.

### LRA estimates of distance-weighted plant abundance for 25 taxa, AD 1685–2010

The LOVE-estimated relevant source area of pollen (RSAP) for the three small sites over the study period varies between 1300 m and 1700 m radius around each site, which is in the range of the forward-simulated RSAPs found by Hellman et al. (2009a,b) for the mid and late Holocene vegetation/ecological setting of southern Sweden. Below, the LRA-LuVs with error estimates equal or larger than the LRA-LuV value are considered not to differ from zero.

**Figure 3:**
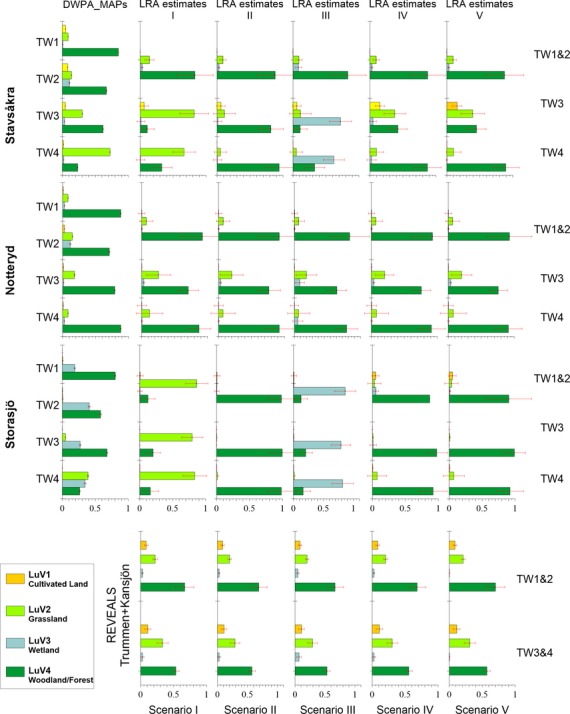
Upper panel: Landscape Reconstruction Algorithm (LRA) estimates of local distance-weighted (DW) plant abundance (DWPA) (in proportion) for four land-use/vegetation categories (LuV 1–4) and five scenarios (I–V, right columns) compared to the DWPA (in proportion) for the same four LuVs extracted from historical maps (DWPA_MAPs) (left column) at Stavsåkra (upper row), Notteryd (middle row), and Storasjö (lower row) for the four studied time windows: AD 1950–2010 (TW1), AD 1925–1950 (TW2), AD 1825–1925 (TW3), and AD 1700–1825 (TW4). Scenarios I to V of attribution of pollen taxa to LuVs are as follows: I. *Calluna* in grassland (LuV2); II. *Calluna* in woodland/forest (LuV4); III: *Calluna* in wetland (LuV3); IV: *Calluna* excluded; and V: *Calluna* and Cyperaceae excluded. Lower panel: REVEALS estimates of regional vegetation abundance using the pollen records from the two large lakes Trummen and Kansjön for the same time windows and scenarios of pollen taxa attribution to LuVs.

**Figure 4:**
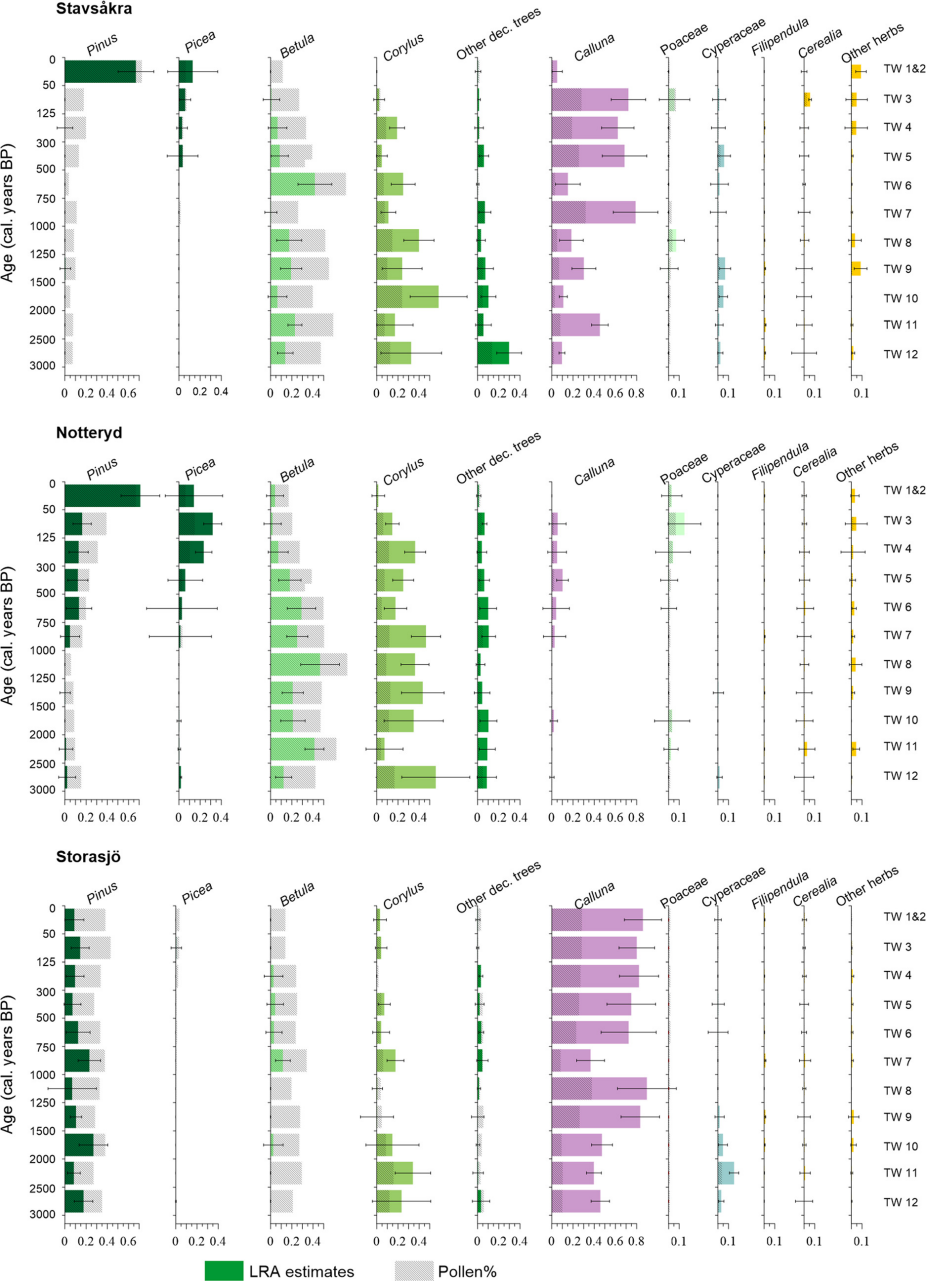
Pollen percentages and pollen-based Landscape Reconstruction Algorithm (LRA) estimates of distance-weighted vegetation abundance for individual taxa or groups of taxa at the three small sites Stavsåkra, Notteryd, and Storajö. “Other deciduous trees” include *Quercus*, *Ulmus*, *Tilia,* and *Fraxinus*, “Cerealia” all cereals (*Triticum* type, *Hordeum* type, *Avena* type and *Secale cereale*), and “Other herbs” include Compositae Subfamily Cichoroidae, *Plantago lanceolata*, *Potentilla* type, *Ranunculus acris* type, Rubiaceae, and *Rumex acetosa* type.

The LRA-LuVs (Fig. 3) and LRA-taxa/-group of taxa (Fig. 4) at Stavsåkra indicate that *Calluna* (60% cover) was dominant in the local vegetation until AD 1870 (TWs 3 and 4), while *Picea* was present locally first around AD 1870 and increased from <10% to 15% AD 1950, and *Pinus* appears and was dominant (65%) first around AD 1950 (TW2). In contrast, *Corylus* (17%) and *Betula* (5%) were still relatively common around AD 1700–1800 (TW4), but were very rare or absent around AD 1870 (TW3). At Notteryd, *Betula* and *Corylus* were dominant until ca. AD 1700 (*Betula*) and ca. AD 1950 (*Corylus*). *Pinus* occurred regularly from ca. AD 1250 (15%) and strongly increased from AD 1950, while *Picea* was first present locally from ca. AD 1700, reached 30% ca. AD 1875, and was probably replaced locally by planted *Pinus* AD 1950. The other deciduous trees (*Quercus*, *Ulmus*, *Tilia,* and *Fraxinus*) occurred until early 20th century. *Calluna* was rare or absent locally during the last three millenia. At Storasjö, *Calluna* (80%) strongly dominated the landscape throughout the study period together with *Pinus* (subdominant 10–15%). Ca. AD 1700–1800 (TW4) *Fraxinus* was relatively abundant (25%), while it was absent from AD 1900 (TWs 3 and 2).

The differences between the LRA-LuVs and the pollen percentages (Fig. 4) are particularly important for *Calluna*, *Betula*, *Fraxinus*, *Pinus*, *Corylus*, and *Picea*. In comparison to their LRA-estimated abundance, *Calluna*, *Corylus*, *Picea*, *Fraxinus,* and *Salix* are underrepresented in pollen percentages, whereas *Pinus* and *Betula* are overrepresented in pollen percentages.

### LRA estimates of local plant composition versus REVEALS estimates of regional plant composition

The REVEALS estimates for the two time periods TW1&2 (AD 1950–2010) and TW3&4 (AD 1685–1906) (fig. 3 in Appendix S1) show that the cover of *Fagus*, *Picea*, *Betula*, Poaceae, and Cyperaceae is higher in the regional vegetation than in the local vegetation at Stavsåkra, Notteryd, and Storasjö, while the cover of *Calluna* is much lower in the regional vegetation than in the local vegetation at Stavsåkra and Storasjö. At Notteryd, *Calluna* is rare or absent. The cover of *Corylus* (TW4) and *Pinus* (TW2) is lower in the regional vegetation than in the local vegetation at Stavsåkra and Notteryd. The unchanged abundance of *Pinus* through time is a characteristic of both the REVEALS regional vegetation and the LRA local vegetation at Storasjö.

## Discussion

### Test of the LRA reconstructions and sources of errors

Sources of error in both map- and LRA-vegetation data and their implications for the testing of the REVEALS and LOVE models are discussed in earlier publications (e.g., Hellman et al. 2008a,b; Nielsen et al. 2012; Overballe-Petersen et al. 2013; Fyfe et al. 2013; Mazier et al. in press). They may be related to the number and distribution of sites, size and type of accumulation basin, pollen productivity estimates, fall speed of pollen, size of the pollen counts, and chronological control of the pollen counts. In the present study, pollen counts are <1000 for Stavsåkra and Storasjö TWs 1–3 (Table 2), which may explain the larger error estimates of the LRA reconstruction compared to the REVEALS reconstruction (Fig. 3, and fig. 1 in Appendix 3). The chronologies at the three sites (Olsson et al. 2010; Cui et al. 2013; Cui 2013) have a precision of ca. ±50 years, and the time resolution of the pollen counts is low. It implies that the LRA estimates span over longer time periods than the maps’ TWs.

The sources of error in the map-LuV data relate primarily to the interpretation of the original maps and attached descriptions in terms of LuV categories and plant composition, and the harmonization of LuVs between maps. The cover of grassland (LuV2) and wetland (LuV3) might be underestimated in favor of woodland /forest (LuV4) because it is not always specified whether part of the outland included grassland and whether wetlands were wooded or open; therefore, the outland and wetlands might be wrongly classified as woodland/forest in some cases. This is most probably the case for some of the historical maps for TW3 at Storasjö where the large woodland/forest patches in the southern and northeastern slices of the 3-km-radius circle should certainly include wetlands as it is the case for TW2 and TW4. Also, woodland/forest might be underestimated in favor of grassland because “meadows” (term used in the historical documents) without specification on whether they were wooded or not were classified as grassland. Further, the LRA-reconstructed LuV cultivated land comprises only cereals (*Cerealia* [cereal] type, *Hordeum* [barley] type, *Triticum* [wheat] type, *Secale cerealia* [rye]), as these are the only cultivated plants for which PPEs are available. Therefore, the LRA-LuV for cultivated land may often underrepresent the actual cover of cultivated land.

When comparing the maps’ vegetation data (map-LuVs) with the LRA estimates (LRA-LuVs), one should remember that the vegetation data extracted from the historical maps do not always represent the entire surface of the 3-km-radius circle around each site and, therefore, might be biased toward the landscape characteristics of a particular area of the studied landscape. This should be taken into account in the interpretation of the comparison between LRA estimates and the vegetation data from the historical maps. Inspection of the maps presented in Figure 2 helps evaluating the probable reliability of the vegetation data for the entire surface. This issue may have consequences on the results mainly for the site Storasjö at the times of TWs 3 and 4. The eastern part of the 3-km-radius circle around the site is the only area that is characterized by larger surfaces of cultivated land in the maps for TWs 1 and 2, and there is no data for that same area at TWs 3 and 4. Therefore, the vegetation data for TWs 3 and 4 might include too little cultivated land (and grassland for TW3). This would explain that the LRA estimates of cultivated land are slightly higher than the percentage cover from the maps, although the difference is minimal in view of the very low percentage of cultivated land at this site in any case. At Stavsåkra, there is one area in the northeastern part of the 3-km-radius circle that is woodland/forest-dominated in the maps for TW1 and 2. That same area is missing in the map data for TW 3 and 4. Therefore, the woodland/forest fraction within the circle might be underestimated for these two TWs. However, it is clear that other areas at Stavsåkra that are dominated by woodland/forest in the 20th century maps (TW1 and 2) are characterized by the occurrence of more or less large grassland patches in the 18th–19th century maps (TW3 and 4); this is particularly clear in the north-northwestern slice of the circle.

In spite of the sources of error and draw backs discussed above, there is a reasonably good agreement between the map-LuVs and the LRA-LuVs, except in some of the cases characterized by high pollen values of *Calluna* (Fig. 3). All scenarios of LRA-LuVs at Notteryd are in strikingly good agreement with the map-LuVs for woodland/forest and grassland. For wetland, the agreement is best when *Calluna* is included either in woodland/forest or grassland, except for TW2 for which there is no good agreement, which is explained by the fact that there is not enough pollen data to distinguish between T1 and T2. The cultivated land has a too low cover (except in the map data of TW2) for a meaningful model-data comparison.

At Stavsåkra, the best matches are found in scenarios II, IV and V for TW1, III for TW2, II and IV for TW3, and I for TW4, although some discrepancies exist. Most of the discrepancies may be explained by the attribution of *Calluna* to a single LuV. If *Calluna* was growing mainly in one of the LuVs, the best match will be found in the scenario with *Calluna* attributed to that LuV, while there will be poor matches in the other scenarios. Such poor matches are found for TW2 (scenario III) and TW4 (scenario I), which suggests that *Calluna* probably was growing primarily in wetland (TW2) and grassland (TW4). In TW3, *Calluna* might have grown mainly in woodland, but likely also in grassland and wetland. The latter interpretation would explain why the best match in this case is found for scenario IV (*Calluna* excluded). If *Calluna* was growing in all three LuVs, and was not dominant in any of the LuVs, the exclusion of *Calluna* would not influence significantly the proportions of the three LuVs.

The best matches between the map-LuVs and LRA-LuVs at Storasjö are found in scenario IV (*Calluna* excluded) for TW1 and TW2. There are no good matches for TW3 and TW4 (Fig. 3). These results suggest that in all cases Calluna was common in more than one LuV. *Calluna* was probably growing in two LuVs in TW1 (mainly in woodland/forest (scenario II) but also in wetland (scenario III)), and in TW2 (both in woodland/forest and wetland). In TW3 and TW4, it may have grown in three LuVs: mainly in woodland/forest [scenario I]), but also in wetland (scenario II) and grassland (scenario I) in TW3, and in woodland/forest, wetland, and grassland in TW4. However, excluding *Calluna* does not produce a better match, because if *Calluna* is the major pollen type representing one or two LuV(s) (wetland and grassland in particular), excluding it will imply an underrepresentation of that (those) LuV(s) and an overrepresentation of the remaining LuVs in the LRA reconstruction.

The fact that *Calluna* can grow in different vegetation types in the study area has long been a difficult issue in the interpretation of pollen data (percentages as well as pollen accumulation rates) in terms of vegetation and cultural landscape history (e.g., Gaillard et al. 1994; Regnéll et al. 1995; Lagerås 2000; Gaillard 2013). The interpretation of both pollen percentages and LRA estimates of taxa such as *Calluna* will always require other sources of information for a more precise interpretation (e.g., the use of insect (Coleoptera) remains and archeological data in Cui et al. 2013). Cyperaceae and Poaceae are also plant taxa that may grow in all three vegetation types. In this study, these pollen taxa are not a problem (low pollen percentages), but it is recommended to perform alternative LRA runs in cases with high pollen values.

**Figure 5:**
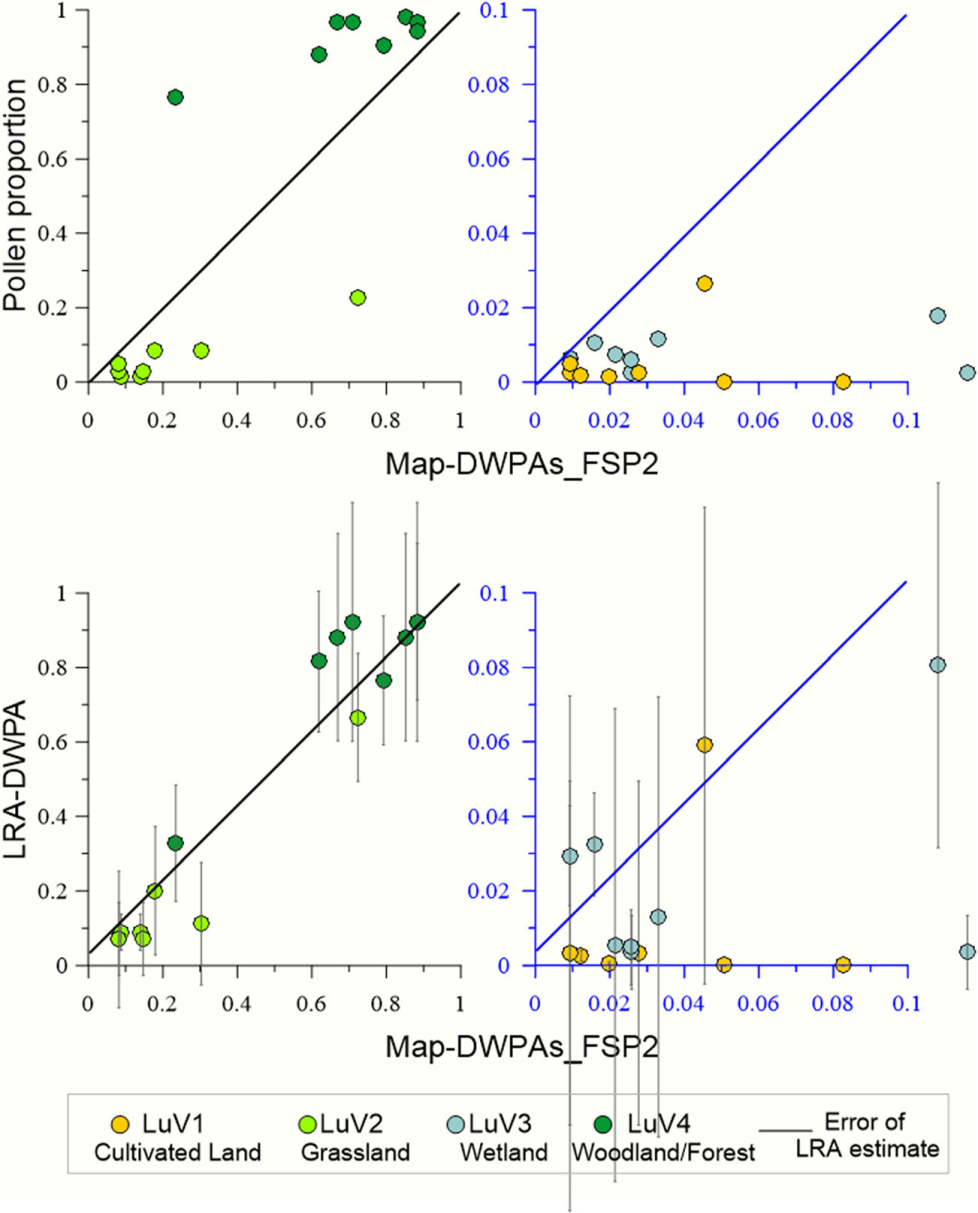
Relationships between distance-weighted plant abundance (DWPA) for four LuVs extracted from the historical maps (map-DWLuVs) and (1) pollen proportions for the same LuVs (upper graphs) and (2) the best scenarios of LRA-DWLuVs (with their error estimates) from Stavsåkra and Notteryd (lower graphs), that is Stavsåkra: *Calluna* growing in woodland/forest (TW1 – AD 1950–2010 and TW3 – AD 1825–1925), *Calluna* growing in wetland (TW2 – AD 1925–1950), and *Calluna* growing in grassland (TW4 – AD 1700–1825); Notteryd: *Calluna* growing in woodland/forest for all TWs.

The plots of the relationships pollen%/map-DWLuVs and LRA-DWLuVs/ map-DWLuVs for the best scenarios at Stavsåkra (see above) and for scenario II at Notteryd (Fig. 5) indicate that the LRA reconstruction corrects for the biases of pollen percentages for both grassland and woodland/forest in all four time windows. Only two values of LRA wetland are different from zero, and in those cases, the LRA provides a reasonable correction of the pollen percentages. All values of LRA cultivated land do not differ from zero; therefore, the LRA correction of cereal pollen percentages cannot be evaluated properly in this study.

There are so far two other tests of the LRA’s performance in Europe: (1) in the Åboda area (Småland Uplands) ca. 25 km W of Stavsåkra and Trummen (Fredh 2012; Mazier et al. in press; Fig. 1) and (2) in eastern Denmark north of Copenhagen (Overballe-Petersen et al. 2013). The test performed in Denmark differs from those in Sweden primarily because of major differences in the type of historical land-use/vegetation data, and the categorization of historical and pollen data. In the test by Mazier et al. (in press), the LOVE estimates were shown to be in reasonable agreement with the data extracted from the cadastral maps. A detailed comparison of Mazier et al.’s results with ours is not possible because of several differences in the methods used. Unforested land and forest/woodland were categorized as “meadow” and “outland”, respectively, in the group of old maps, and as grassland and woodland, respectively, in the group of recent maps. Further, “meadow,” “grassland,” “outland,” and “woodland” were assigned different pollen types. In contrast, we did not distinguish a “meadow” LuV and “outland” LuV, and categorized “outland” as either grassland or woodland using information from written documents associated to the maps. Moreover, Mazier et al. (in press) attributed *Corylus* to “meadow” or “grassland” and *Juniperus* (juniper) to “outland” or “grassland” depending on the time window, and *Calluna* to wetland. We consistently ascribed *Corylus* to “woodland” and *Juniperus* to “grassland” in all time windows, and *Calluna* to three alternative LuVs.

### Insights on the last three centuries of landscape history and diversity in central Småland

The comparison between LRA-LuVs and map-LuVs discussed above indicates that LRA reconstructions provide a reasonable picture of the cover of major plants and landscape/land-use units over the past three centuries. Therefore, the information provided by the historical maps can be discussed in the temporal context of the pollen-based LRA vegetation reconstructions. Combining the two analyses (historical maps and LRA reconstructions), we can draw new insights on the essential role played by landscape characteristics and land-use practices over several millennia in the development and maintenance of biodiversity in the hemiboreal zone of southern Sweden. Although LRA estimates of vegetation cover at the three sites are available for the entire Holocene (the last eleven millennia), we chose here a perspective of three millennia because it is the time during which humans started and increased their use of and influence on the landscape/vegetation of our study region (e.g., Greisman and Gaillard 2009; Olsson et al. 2010; Cui et al. 2013).

#### Regional versus local vegetation characteristics

The decreases through time of *Calluna*, *Betula* and *Corylus*, and the related increase in *Picea* (mainly planted) from the end of the 19th century are trends in vegetation development that are found at both the local scale at Stavsåkra and Notteryd (LRA reconstruction) and the regional scale (REVEALS reconstruction) (fig. 4 and fig. 3 in Appendix S1). The significantly higher abundances of beech and spruce in the REVEALS regional vegetation in comparison to their abundance in the local vegetation are most probably due to a higher abundance of beech around and south of Växjö (area of Trummen) and of spruce (natural or planted) around and N of Växjö (area of Kansjön). Nonetheless, the pollen percentages of spruce for modern time (AD 1950–2000) at Trummen (regional), Stavsåkra, and Notteryd (local) are comparable. There are also higher abundances of *Quercus*, *Tilia*, *Ulmus*, *Alnus*, *Betula*, Poaceae, and Cerealia in the REVEALS regional vegetation than in the LRA local vegetation; this is obviously due to higher abundances of these taxa (except *Betula* and Poaceae) in the area of Trummen than elsewhere in central Småland, which is a particularity of the Växjö area as already pointed out by Hellman et al. (2008a,b) and Cui et al. (2013). However, because the taxa mentioned above, except *Betula*, have very low representations (less than 2%) in both the regional and local vegetation at all sites, the influence of the pollen record from Trummen on the LRA reconstructions of local vegetation is very small. The high pollen percentages of pine at Trummen and Kansjön are comparable; thus, the REVEALS-estimated regional abundance of pine should be reasonable, while the high REVEALS estimates for spruce might be biased by the high pollen percentages at Kansjön. This may also imply a too low estimate of spruce at the local scale.

The lower representation of heather in the REVEALS regional vegetation compared to its share in the LRA local vegetation at both Stavsåkra and Storasjö indicates that *Calluna* heaths did not represent more than 4–5% of the regional landscape, while it could be locally dominant. Moreover, pollen from woodland ground plants may be under-represented in pollen records from large lakes, as their dispersal is hampered under the tree canopies (in the “trunk space”), a phenomenon that is known since the studies of Tauber (1965) and Andersen (1970). The site Notteryd, with no or very little cover of *Calluna*, is an example where *Calluna* was rare or absent locally.

#### History of landscape diversity in central Småland and implications on habitat and species diversity

A comparison of our study (Växjö and Storasjö areas) with the study in the Åbodasjön area (Mazier in press Fig. 1) illustrates the diversity in local vegetation characteristics over space and time. The Åboda study covers the time period AD 1800–2008 and provides vegetation reconstructions for areas ranging between 0.35 and 4.4 km in radius around the two small lakes. There are clear similarities between the REVEALS reconstructions of the regional vegetation in the Åboda study and ours (Mazier et al. in press/our study). Spruce is dominant (25–35%/35%) ca. AD 1950, and pine subdominant (20%/15%) throughout the period. Poaceae (10–15%/20%) and cereals (10–20%/10%) are the most abundant herbs, and hazel decreases from ca. AD 1900 and heather from ca. AD 1940/before AD 1950. Spruce, beech (ca. 2%), and cereals were more abundant, while hazel and heather were less abundant in the REVEALS regional vegetation than in the LRA local vegetation.

The Åbodasjön site is distinguished by a high abundance of hazel and other broad-leaved trees. Grasses and juniper are relatively abundant around AD 1820–1920. In contrast, the Lindhultsgöl site is characterized by a dominance of pine and birch (except 2000–2008), and heather represents >10–25% of the vegetation until AD 1960. It is well known from historical documents that spruce plantations became increasingly common in southern Sweden – and the province of Småland in particular – from the beginning of the 20th century (Eliasson 2002) Lindbladh et al (in press). In our study (Fig. 3), we record a decrease in deciduous woodland related to an increase in conifers (pine and spruce) at Stavsåkra and Notteryd from the “Storskifte” time (TW4, 18th century) to the “Enskifte” period (TW3, 19th century), and an almost total absence of deciduous trees and a high cover of pine in TW1&2 (1950–2010). A similar trend is documented at Lindhultgöl (first with pine from AD 1920, then with spruce from AD 1960) and Åbodasjön (spruce from AD 1920) (Fredh 2012). The general decrease in cover of wetland during the 20th century is best documented in our study by both the LRA reconstructions and the map data, especially at Storasjö (Fig. 3). It is clearly related to drainage for the improvement of modern forestry. There is also a decrease in wetland at the Åbodasjön site around AD 1900–1940 (drainage to increase the cropland area) and at Lindhultsgöl from AD 1920 to the present (drainage for forestry).

The two studies together provide an interesting collection of local land-use and vegetation histories of the 19th century (Fig. 4): Lindhultsgöl and Storasjö are most similar with pine-birch woodland and ground vegetation dominated by heather (our interpretation), although hazel and birch are abundant at Lindhultsgöl, while pine and heather are dominant at Storasjö. Åbodasjön, Notteryd, and Stavsåkra have a similar woodland history, with more broad-leaved trees than the other two sites, but there is no development of *Calluna* heath neither at Åbodasjön, where juniper is characteristic instead, nor at Notteryd where hazel is dominant. These differences might have their explanation in the land-use history previous to the 19th century at the five sites (Fig. 4). At Åbodasjön, juniper became as common as during the 19th century already ca. AD 1600 as grazing land developed at the expense of spruce woodland (Fredh 2012). At Lindhultsgöl, juniper increased mainly at the expense of pine, but it had relatively low values already from AD 1700, to become rare from AD 1840. It might indicate that grazing decreased earlier around Lindhultsgöl than Åbodasjön (our interpretation). The long-term history is very different at Stavsåkra where *Calluna* heaths established already ca. 1000 BC at the expense of birch and broad-leaved trees (Cui et al. 2013); *Calluna* heaths became dominant from AD 1000 and first decreased in cover after AD 1870. At Notteryd, hazel was dominant already from ca. 1000 BC (3000 BP). The high percentage cover of hazel until recently and also birch until ca. AD 1500 are ascribed to the development of hazel woodland because of humans favoring and managing this species for wood collection and woodland grazing.

Further information on land-use and landscape characteristics through time are provided by insect data from both Stavsåkra and Storasjö. Olsson and Lemdahl (2009, 2010) showed that insect species strictly confined to *Calluna* heathland, in particular *Bradycellus ruficollis* (a ground beetle), and dung beetles were common at Stavsåkra from 1200 BC to AD 1400, decreased after AD 1400 and disappeared after AD 1800 (today extinct in the region). These beetle species were also common at Storasjö from 900 BC to AD 1800, although occurring with an abundant saproxylic insect fauna feeding on both deciduous and coniferous wood, which was not the case at Stavsåkra. The diversity of the insect fauna decreased considerably after AD 1800 at both sites. The insect, pollen, and LRA data together suggest that grazed *Calluna* heaths were dominant in the landscape at Stavsåkra and managed as such until ca. AD 1825 (end of the Storskifte). At Storasjö, pine woodland was characteristic of the landscape during the entire Holocene. It was used more intensively for cattle grazing from at least 900 BC, which lead to the formation of patches of *Calluna* heath that maintained until ca. AD 1800 (see also map for TW4, Fig. 2). The latter is supported by dendrochronologically dated fire scars on pine attributed to human-caused burning ca. AD 1400 – 1800 (Wäglind 2004; Marlon et al. 2010). Moreover, remains of wood constructions to dry hay dating from the early 20th century were found in the pine forest, indicating that the woodland was more open and also used for hay making (Wäglind 2004). From the Enskifte-Lagaskifte period, after AD 1800, cattle grazing and fire decreased or ceased, which implied that patches of open heaths decreased, but *Calluna* remained an important element of the pine woodland (fig. 1 in Appendix S3). Official fire suppression occurred from AD 1850 in southern Sweden (Niklasson et al. 2002; Lindbladh et al. 2003).

## Conclusions

Comparison of LRA-LuVs and map-LuVs over the last three centuries provides a useful test of the performance of the Landscape Reconstruction Algorithm (LRA) to estimate the cover of land-use/vegetation units in the past. Given that the sources of errors are numerous in both the extraction of land-use/vegetation data from historical maps and the LRA reconstructions, the agreement between the two is strikingly good in many of the scenarios. Therefore, we are confident to suggest that the performance of the LRA is reasonable in our study area, and the modeling approach can be used to reconstruct the cover of land-use/landscape units such as woodland, wetland, and grassland at the local spatial scale.

A comparison of our test with two other tests of the LRA in NW Europe suggests that careful harmonization of LuVs between different historical maps and between maps and LRA reconstructions, as performed in our study, may improve testing the LRA, in particular for LuVs woodland/forest, grassland, and wetland. Our study also shows that LRA reconstructions using the multiple scenario approach for the attribution of pollen taxa to LuVs and, in the case of Swedish historical sources, the careful use of the notes attached to the maps greatly help testing the LRA performance and identifying causes behind discrepancies between LRA- and map-LuVs.

The historical maps indicate that the landscape diversity and evenness decreased dramatically around the three study sites over the last 3 centuries. Our study together with the investigation by Mazier et al. (in press) show that, over the three last centuries, each of the five small sites had its specific land-use/landscape history, implying five different landscape mosaics in each time window, that is a multitude of landscape mosaics over time and space. A similar interpretation was already proposed by Lindbladh et al. (2000) for the last 3k years based on pollen percentages from sites of various sizes in southern Sweden. Moreover, our study illustrates that each site’s recent history has its roots in the long-term local land-use history over the last two to three millennia. Similar conclusions were put forward by Hultberg et al. (2014) based on pollen data and LRA reconstructions from three sites located some 50 km east of our study region. Here, we complement the observation by Lindbladh et al. (2000) and conclusions of Hultberg et al. (2014) with the study of historical maps and related pollen-based quantitative reconstructions of plant abundance. We argue that the diversity in landscape content during the last three centuries, including the remaining, modern diversity can be explained by the long-term land-use history over one to several millennia at each site, but that most of the past diversity was lost over the two last centuries through the agrarian reforms from the 18th century and modern land-use practices. These reforms resulted in a drastic decrease in area and fragmentation of wetlands, in grazing land and hay meadows already in the early 19th century, and in their increasing rarity after AD 1950. Over the last two centuries, they decreased to less than a half of their former cover, while the area of conifer woodland/forest (spruce in particular) at least doubled. Pine and spruce were increasingly planted in the grazed outland and infield meadows, and modern forestry rapidly replaced agriculture and stock farming in the region. Landscape diversity and evenness decreased drastically, implying a dramatic loss of habitat and species diversity (e.g., beetles). Such development can only be stabilized (and possibly reversed) by major changes in landscape and forest management and conservation measures, including appropriate management of still existing hay meadows, *Calluna* heaths, and broad-leaved and mixed conifer woodland/forest, as well as restoration of such environments within the spruce-dominated forest landscape in order to counteract the still ongoing fragmentation of grassland (grazed pastureland and hay meadows) and *Calluna* heath habitats.

This study illustrates that an understanding of the landscape as a whole, its characteristics and changes over time and space, is as essential as the investigation of local changes at the modern sites identified as biodiversity hot spots as undertaken by Niklasson et al. (2002), Lindbladh et al. (2003), Lindbladh et al. (2008), Hannon et al. 2010; and Hultberg et al. (2014). It provides the historical broad context in which those sites have developed. Modern biodiversity hot-spot sites are remnants of former large cultural landscapes and are “refuges” for species that were distributed over much larger areas and in a higher variety of land-use/landscape types in the past. Today, some of these species might be “living dead”, still existing species that do not have the necessary environment for a long-term survival in the future. For the maintenance of these endangered species and adequate strategies for conservation, it is a necessity to understand their history at the site but also, and perhaps most importantly, in the entire regional landscape.

## Supporting Information

**Appendix S1** Historical maps, pollen data and methods
