# Supplementary material for: Historical land-use and landscape change in southern Sweden and implications for present and future biodiversity
Source: Ecol Evol. 2014 Sep 1;4(18):3555–70. doi: 10.1002/ece3.1198 (PMC4224531; doi:10.1002/ece3.1198)
Supplement: Supplementary file 1 — Appendix S1 Historical maps, pollen data and methods [file ece30004-3555-sd1.pdf]

APPENDIX S1

DETAILED INFORMATION ON HISTORICAL MAPS, POLLEN DATA AND METHODS

Historical maps and harmonization of the information between maps and with the pollen-based reconstructions of land-use/vegetation cover

Extraction of vegetation data from historical maps

The size of the area for which land-use/vegetation data is extracted from the historical maps need to be at least as large as the Relevant Source Area (RSAP sensu Sugita, 1994) of the study sites. As the Landscape Reconstruction Algorithm (LRA, see end of Appendix under "LRA reconstructions") estimates of RSAP over the studied time windows are ranging between 1.2 and 1.7 km radius, we chose to extract vegetation data in a 3 km-radius area around each study site (pollen sampling site). All available modern and historical cadastral maps for those areas were collected from the Swedish Land Survey and grouped into four time windows (TW) based on knowledge of the land reforms in southern Sweden (Table 1). Only the modern maps (TW1 'Terrain Map' and TW2 'Economic Map') provide 100% cover of the study area. The maps from the reform periods (TW3 'Enskifte-Lagaskifte' and TW4 'Storskifter and Older') often document only one village and its surrounding land. All existing maps were thus combined for each of the TWs 3 and 4 to obtain the largest possible cover.the study areas.

Each original map was scanned. ArcGIS was used for rectification, digitization, visualization and extraction of the vegetation information. Rectification of the historical and economic maps was performed by coupling geo-reference control points (such as cross roads, lake and administrative borders) to a geo-referenced modern base map using the coordinate system

SWEREF 99. The reform maps include detailed information on land-use/vegetation units of generally high technical and geometric quality (Örback, 1998), which allows rectification with a relatively low degree of error.

Table 1 Historical maps within a 3 km-radius area around the three study sites.

Time Windows	TW1&2	TW3	TW4	
	Modern	Enskifte-Lagaskifte 1825-1922	Storskifte 1772-1825	Older 1685-1740
**STAVÅKRA**	Economic map (1950); Terrain Map (2010)	Borlanda (1832); Aby (1848); Tjureda (1850); Aby (1869); Eknaholm (1869); Stavsåkra (1906)	Borlanda (1780); Tjureda (1780, 1820)	Geometrisk (1715); Ekan (1698,1740)
Coverage of Maps (%)	100%	75%	24%	10%
**NOTTERYD**	Economic map (1950); Terrain Map (2008)	Näset (1852); Notteryd (1860); Älmåsen (1872)	Älmåsen (1760); Tofta (1793); Gårdsby (1797); Inagor (1805,1821); Utmark (1805); Vikensved (1817)	Sandsbro (1725)
Coverage of Maps (%)	100%	12%	63%	11%
**STORASJÖ**	Economic map (1950); Terrain Map (2008)	Lillasjö (1844); Hökaskruv (1853); Djupgöl (1856); Bihult (1858)	Tonaberg (1786)	
Coverage of Maps (%)	100%	77%	59%	0%

Harmonization of land-use/vegetation classes

The harmonization of the maps' original land-use/vegetation classes (LuVs) implied assumptions based on expert knowledge of local vegetation and land-use history in the study area. There is no distinction of different cultivated crops on the maps because cultivation followed various rotation systems. Therefore, "Cultivated land" (LuV1) is all land that was cultivated with crops (mainly cereals and potatoes), or fodder/lay (mainly grass or leguminous plants such as *Medicago sativa* (alfalfa or lucerne)). "Cultivated land" is thus not necessarily cereals and could periodically lie fallow. Because differentiation of open-land classes is not identical between individual maps, open pastureland (sometimes called "Outland") could not

be separated from hay meadows and other unspecified open land areas. Therefore, all classes were grouped into a single class “Grassland” (LuV2), however carefully taking into account the written information related to the maps. Some of the meadow classes might have been half-wooded land as wooded hay meadows were a common feature of the traditional cultural landscape of southern Sweden (Emanuelsson, 2007; Berglund, 1991; Gaillard, 2010), but the meadow class was more often specified as “meadow without tree”. Therefore, “meadows” were classified as “Grassland”. We combined bogs and fens with other wetland classes such as mowed meadow on wet soils and unspecified wetlands into a single “Wetland” class (LuV3). As deciduous woodland was not always separated from conifers, all classes with tree cover were grouped into a “Woodland/Forest” class (LuV4). It includes managed forests/tree plantations as well as non-managed woodland as these are not separated in the maps. It also includes the class “Outland” documented in some maps, representing woodland areas that were generally part of the commons of villages, outside the “infields” with cultivated fields and hay meadows (Berglund 1991). In these maps, “Outland” that was not wooded is sometimes illustrated by some generalized tree symbols scattered over the area and specified in related notes and tables as “Outland without trees”. Those “Outland” areas where classified as “Grassland”.

Assignment of pollen types to the maps' LuV classes

The Landscape Reconstruction Algorithm (LRA, see end of Appendix under “LRA reconstructions”) reconstruction requires a number of known parameters of which pollen productivity estimates (PPEs) are particularly difficult to obtain. In this paper we use 25 pollen taxa for which PPEs are available (Mazier et al., 2012; Cui et al., 2013) (Table 3). As the LRA estimates of Land-Use/Vegetation classes (LuVs) (LRA-LuVs) will be compared with LuV data extracted from historical maps (map-LuVs), and because the LuVs are

characterised by groups of plant taxa, the 25 pollen taxa used for the LRA reconstructions have to be assigned to the LuVs.

In this study, *Calluna* (heather) is the most problematic case because it exhibits high pollen counts and percentages (20-30%) at both Stavsåkra (TW3-4) and Storasjö (TW1-4) (see Fig. 3), implying high LRA estimates of its cover in the vegetation (60-65 % at Stavsåkra, 75-85 % at Storasjö). Existing vegetation surveys in conifer woodlands and bogs at Storasjö (Bunting and Farrell, unpublished; survey protocol described in the the Crackles project http://www2.hull.ac.uk/ science/crackles.aspx) and in grazed heaths and grasslands in the provinces of Skåne, Blekinge, Småland and Halland (unpublished, survey method described in Gaillard et al., 1992) provide some estimates of the modern cover of *Calluna* for southern Sweden (Table 2). The highest percentage covers are found in grazed dry heaths (maximum 88%) and grassland (maximum 40%) and the lowest in woodland (maximum 12 %) and bog (< 5%). Note, however, that the surveys at Storasjö are not representative of all conifer woodlands and bogs in southern Sweden. *Calluna* may be very abundant in more open conifer woodlands and large peat bogs. However, these surveys indicate that the woodlands and bogs of the Storasjö area are characterised today by a relatively low cover of *Calluna*, and that open grazed heaths in southern Sweden may have 80 to 90% cover of *Calluna*. It also shows that between-site variation in abundance is very high in both grassland and woodland. We assume that *Calluna* was also growing in the three major vegetation types (grassland/heath, wetland, and woodland) in the past and exhibited a similar between-site variation in abundance.

Therefore, we chose to examine three different scenarios of assignment of pollen taxa to the LuVs 2-4, i.e. *Calluna* in (I) Grassland (LuV2), (II) Wetland (LuV3) or (III) Woodland/Forest

(LuV4). We also present scenarios were *Calluna* (IV) and *Calluna* + Cyperaceae (V) are excluded, following the strategy chosen by Nielsen et al. (2012) for Cyperaceae.

Table 2 Mean percentage cover of *Calluna* (heather) with their standard error (SE) in the Land-use/Vegetation categories (LuVs) Grassland, Woodland/Forest and Wetland of southern Sweden. The data are from Gaillard et al. (1992, 1994)* and the Crackles project (unpublished, personal communication from Jane Bunting and Michelle Farrel, Hull University, U.K.) **.

	**Grassland***			**Woodland/Forest****				**Wetland**
Plot number and definition	4	16	1	25	25	25	25	4
	Grazed grassland	Dry heath	Wet heath	Site 1	Site 2	Site 3	Site 4	Bog
Mean % cover with range or SE	16.4	37.6	10.9	1.0±0.4	11.9±2.1	2.2±1.0	3.2±0.7	<5%
	0.1–39.7	3.9–87.7						
Mean % cover in LuVs and SE	**32.3±4.9**			**4.6±0.8**				**<5%**

Below, "*Calluna* scenarios I-V" refer to the five scenarios above. In this study *Salix* (willows) is assigned to Wetland, although the map-wetland unit is primarily open wetland or wetland with little tree cover. However, the high values of *Salix* at Stavsåkra are assumed to be related to the recent afforestation of the bog site with primarily *Pinus* (pine), but also some *Picea* (spruce) and willow. In contrast, *Alnus* (alder) was assigned to Woodland because the maps' woodland unit includes wetlands with trees such as *Alnus*. At Storasjö, two maps of TW3, one in the southern part and the other in the northeastern part of the study area could not be interpreted correctly in terms of open wetland (LuV Wetland) because the maps did not provide that information and characterised the entire areas as woodland, although it is clear that open wetlands did occur both before (TW4) and after (TW2) TW3. However, we did not

want to add an assumption and kept the original description of these historical maps. The latter implies that the covers of the LuVs Woodland and Wetland are probably overestimated, respectively underestimated, in TW3 for Storasjö.

Distance-weighting of vegetation data extracted from historical maps

Distance-weighting of plant abundance (abbreviated DW below) for the map-LuVs requires that vegetation abundance (here Land-Use/Vegetation (LuV) data) in absolute values (cover in m^2) and fall speed of pollen (FSP) for each LuV (LuV-fall speed) are available. Different methods were suggested to calculate fall speed for groups of pollen types (e.g. Nielsen and Sugita, 2005; Mazier *et al.*, in press; in Fredh, 2012). In this study we tested three methods (test described below) and chose to use the map-DWLuVs calculated with the LuV-fall speeds obtained with the method 2, as described below.

Fall speed of pollen for LuVs (LuV-fall speed)

Values of taxon-specific fall speed of pollen are available for the 25 taxa included in the LRA reconstruction (Table 3). Fall speed can be either measured or calculated with the Stoke's law (Gregory, 1973) using size measurements of the pollen grains from the actual plant taxa (e.g. Mazier et al., 2012). As we need fall speed values for LuVs, we have to calculate fall speed for groups of pollen taxa (here assigned to LuVs To do so, Nielsen and Sugita (2005) used the taxon-specific fall speeds and weighted them with the proportion of each pollen taxon in the assemblage of all pollen taxa used in the study before calculating the mean fall speed for the group of pollen taxa assigned to the LuVs in the maps. This method can also be used without weighting the taxon-based fall speeds (our first alternative Alt 1). Mazier et al. (in press, in Fredh, 2012) summed the mean diameter of all pollen types included in a LuV to get the

"taxa-group pollen diameter"). The taxa-group fall speed of pollen was then calculated using the Stoke's law and the "taxa-group pollen diameter" (our second alternative Alt 2). A fourth possible approach is to calculate the "taxa-group pollen diameter" by weighting the diameter of each pollen taxon in the group with its proportion in the pollen assemblage used in the study (comparable to the first approach above, our third alternative Alt 3).

Here we use the three alternative methods (Alt 1-3 above) to assess their effect on the calculation of the taxa-group fall speed of pollen and map-DWLuVs. Method Alt 3 implies different calculations for the five scenarios of pollen taxa attribution and the three time windows (TWs), i.e. 15 different pollen assemblages, therefore 15 different sets of LuV-FSPs (Fig. 1). Methods Alt1 and Alt2 provide very similar results for all five *Calluna* scenarios, four TWs and two sites (results shown only for one site and one TW in Fig. 1). LuV-fall speed is always slightly higher when method 2 is used. The differences between scenarios are due to the alternative *Calluna* assignments to LuVs. The variation in LuV-fall speed is much larger when Method Alt3 is used, both between scenarios and TWs. Moreover, the values are very different from those obtained with methods 1 and 2. This is of course due to the weighting of pollen-type sizes.

Distance weighting plant abundance for LuVs

We extracted absolute LuV data (in m^2) from the four harmonized maps (TW1-4, seeTable 1) in 10-m increments from the edge of the coring site out to 3 km using ArcGIS. The non-pollen-producing areas (NPP in Fig. 2 in the main article), that is lakes, and settlements (villages, towns). Deforested areas/clear cuts were excluded from this dataset. For the time windows TW3 and TW4 with partial documentation of LuVs (10 – 77% of the total 3km-radius area, Table 1), we assumed that the undocumented part of the study area (No

Vegetation Data, NVD in Fig. 2 in the main article) had the same LuV composition than the documented part.

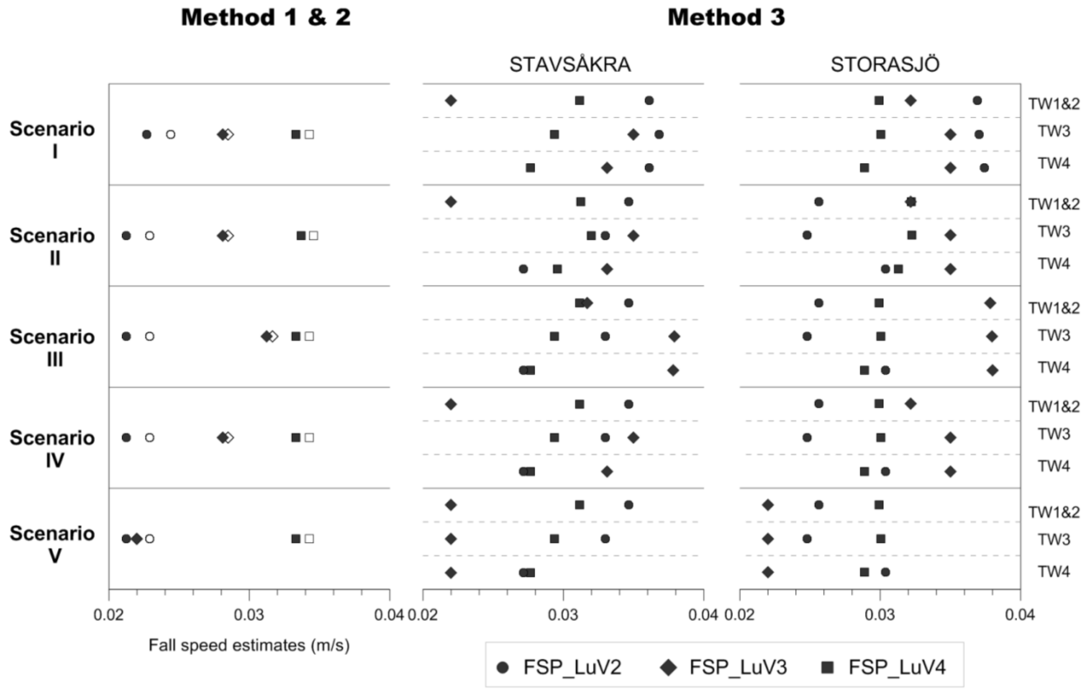

Figure 1: Estimates of fall speed of pollen for LuV2=Grassland, LuV3=Wetland, and LuV4=Woodland/ Forest using three method alternatives (Alt 1-3, see text for method description) for the five *Calluna* scenarios as described in the text above under *"Assignment of pollen types to the maps' LuV classes"*.. Left panel, methods Alt 1 (black) and Alt 2 (white) without weighting the fall speed or pollen size (see text for explanations) for one time window (TW3) and one site (Stavsåkra). Central and right panels, method Alt 3 with weighting of pollen size for both sites and three time windows (TW).

The map-DWLuVs (in proportion) for the time windows TW1-4 and scenarios I-V were calculated using the program DWPA calculator v6.0 (Sugita, 02 May 2012, unpublished) with the map-extracted LuV data at 10m-increments (in m^2) and the three sets of LuV-fall speed obtained with method Alt 1-3 as input data (Fig.2). We used the Prentice's pollen dispersal

model for bogs (Prentice, 1985) and the mean radius of the 3 bog sites as the "common radius".

The differences between the map-DWLuVs using fall speeds Alt 1 or Alt 2 are very small, while the differences are larger between using fall speeds Alt 2 or Alt 3. The maximum differences are found for LuV2 (-12% at Stavsåkra, -7% at Storasjö) and LuV4 (+8% at both Stavsåkra and Storasjö) in scenario I (TW4) (Fig. 2). However, the effect on map-DWLuVs of the method used to calculate the fall speed for groups of pollen taxa is generally not very large. Therefore, the differences in map-DWLuVs wouldn't affect the test of the LRA's performance significantly. However, weighting taxon-specific fall speed of pollen or pollen diameters with the pollen proportions in the pollen assemblages used for the LRA reconstruction appears to be a circular approach. The LRA-estimated and map-extracted DWLuVs should be independent of each other if the LRA reconstructions are to be tested. Therefore, we propose that the method used by Mazier et al. (in press; in Fredh, 2012), here Alt 2, is the soundest one in this case, and we recommend using this method in similar tests.

There are almost no between-scenario differences in map-DWLuVs when FSP2 is used, which is explained by small between-scenario differences in fall speed Alt 2 (FSPs 2) (Fig. 1). There are large between-site differences in map-DWPLuVs when fall speed Alt 2 (FSP2) is used (Fig. 2). In TW4 (AD 1700-1800) Grassland is dominant at Stavsåkra with ca. 74%, whileGrassland, Wetland and Woodland/ Forest have almost equal share (37/37/26) at Storasjö. Woodland/Forest is subdominant at Stavsåkra (23%), and Cultivated land and WEtland have each 1% cover. In TW3 (AD 1800-1900), Woodland/Forest (62%) has increased compared to TW4 at the expense of Grassland; it is now dominant at both sites.

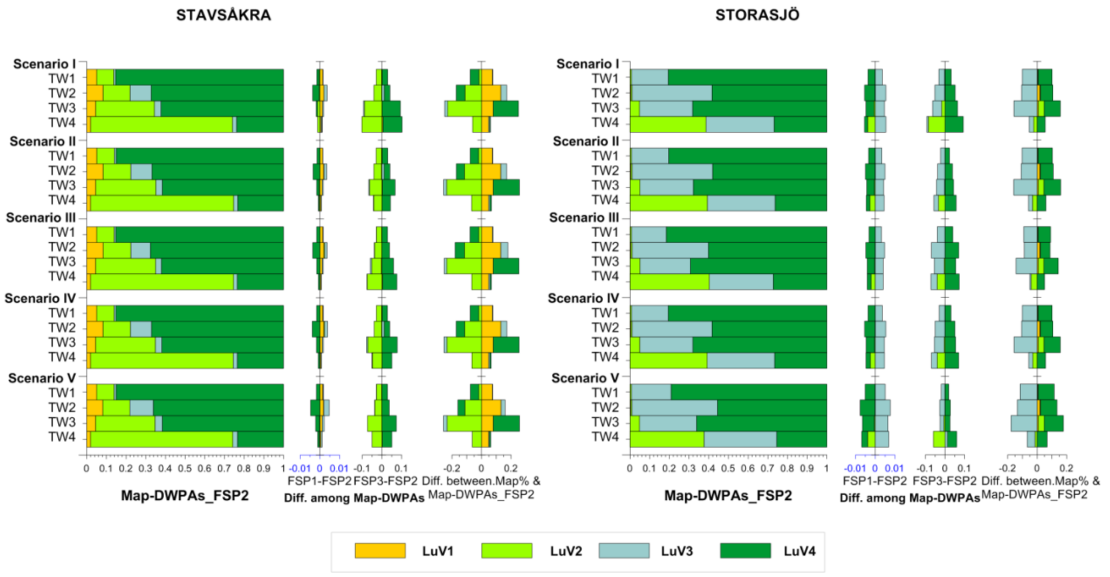

Figure 2 Distance-Weighted Vegetation Abundance of four Land-Use/Vegetation classes from historical maps (map-DWLuVs) at two sites using three different sets of fall speed estimates (abbreviated FSP 1-3), obtained with three alternative methods (Alt 1-3), see text for more explanations). For each site Stavåkra (left) and Storasjö (right), left panel: map-DWLuVs calculated with FSP2 in proportion (the sum of all DWLuVs is equal to 1); right panel: differences between DWLuVs calculated with i) FSP1 or FSP2 (FSP1 minus FSP2, left), and ii) FSP2 and FSP3 (FSP3 minus FSP2, right). LuV1=Cultivated land, LuV2=Grassland, LuV3=Wetland, LuV4=Woodland/Forest.

Cultivated land is slightly better represented (2%) than earlier at Stavsåkra. The share of WEtland is larger AD 1950 than earlier at both sites, and Cultivated land has increased to 4% at Stavsåkra. The modern landscape (AD 2008-2010) is characterised at both sites by a large dominance of Woodland/Forest (84% at Stavsåkra, 79% at Storasjö). Grassland is almost

inexistent at Storasjö, while it still represents 8% of the landscape at Stavsåkra. Cultivated land (2%) has decreased slightly compared to TW2.

Information on the pollen records, Pollen Productivity Estimates (PPEs), Fall Speed of Pollen and the LRA reconstructions

Pollen records

The chronologies for the pollen counts used in this study are from Olsson *et al.* (2010) for Stavsåkra and Storasjö (LRA runs), Cui (2013) for Notteryd (LRA run), and Cui *et al.* (2013) for Kansjön and Trummen (REVEALS runs). Detailed information on the pollen data used and discussed in the main article is presented in Table 1 of the main article and Figure 3 of this Appendix. Figure 3 presents the pollen data in percentages and related pollen-based LRA estimates over the last 3000 years for the three small sites Stavsåkra, Notteryd, and Storasjö. The complete pollen data in percentages over the last 11 000 years are published in Greisman and Lemdahl (2009) (Stavsåkra), Cui (2013) (Notteryd) , Greisman (2009) (Storasjö), and Cui et al. (2013) (Trummen and Kansjön). Because the time resolution of pollen counts is low, there are only few pollen counts that can be compared to the maps for the four time windows (TW1-4). There is only one pollen count in each site that can be compared to TW1 and 2; they are dated to ca. AD 1950 (±50) at Kansjön and Trummen, and ca. AD 1930 ± 50, AD 1940 ± 50, and AD 1935 ± 50 at Stavsåkra, Notteryd and Storasjö, respectively. Therefore, the LRA-estimates are best to compare with TW2 than with TW1.

Pollen productivity estimates (PPEs) and fall speeds of pollen

The LRA reconstruction requires a number of known parameters (Sugita, 2007a,b) of which PPEs are particularly difficult to obtain. In this study we use 25 pollen taxa for which PPEs are available (Mazier et al., 2012; Cui et al., 2013. In this study, we use the fall speeds and PPEs recommended by Hellman *et al.* (2008a and b) for southern Sweden (Table 3).

LRA reconstructions

The possible time resolution of the LRA reconstruction depends of the time resolution of the pollen records. Because of the low time resolution of the pollen records, the REVEALS reconstruction had to be restricted to two time windows, TW1+2 (2 pollen counts) and TW3+TW4 (4 pollen counts), and the LRA reconstruction to three time windows, TW1+2 (1 pollen count per site), TW3 (1 pollen count per site) and TW4 (4 pollen counts for Stavsåkra, 2 pollen counts for Storasjö). The reliability and precision (error estimates) of LRA estimates of distance-weighted plant abundance for the Land-Use/Vegetation categories (LRA-LuVs) tend to increase with the size of pollen counts; theoretically, the precision of the LRA-LuVs should increase with the size of the pollen counts used, i.e. the error estimates of the LRA-LuVs should decrease with increasing pollen counts (Sugita 2007b). Therefore, the LRA-LuVs are expected to be more reliable and precise for TW4 than TWs 1-3 (see pollen counts in Table 1, main article). The LRA-LuVs within the Relevant Source Area of Pollen were calculated by summing up the taxon-based LRA estimates (Fig. 3) of all taxa assigned to a particular LuV using the computer program DWPA.calculator.(version 6.0) provided by Sugita (May 2012, unpublished). The calculation of the error estimates for the LRA-LuVs was achieved using the delta method and Monte Carlo simulation (Stuart and Ord, 1994) (for details, see Mazier et al., 2012). LRA- LuVs were calculated for the four TWs and the five

Calluna scenarios using 25 taxa in scenarios I-III, 24 taxa in scenario IV (*Calluna* excluded), and 23 taxa in scenario V (*Calluna* and Cyperaceae excluded).

Table 3 Pollen productivity estimates (PPE) and fall speeds of pollen (FSP) used in this study (after Mazier et al., 2012). All PPEs and their standard errors (SE) are from southern Sweden, except those with an asterisk that are from Denmark. t=type (pollen-morphological type); Comp.=Compositae; SF.=Subfamily.

Taxa	FSP (m/s)	PPE	SE	Taxa	FSP (m/s)	PPE	SE
Acer	0.056	1.267	0.452	*Picea*	0.056	1.757	0.000
Alnus	0.021	4.200	0.140	*Pinus*	0.031	5.663	0.000
Betula	0.024	8.867	0.134	*Plantago lanceolata**	0.029	0.897*	0.235*
*Calluna vulgaris**	0.038	1.102*	0.054*	Poaceae	0.035	1.000	0.000
Carpinus	0.042	2.533	0.070	*Potentilla*-t	0.018	2.475	0.377
Cerealia-t*	0.060	0.747*	0.039*	*Quercus*	0.035	7.533	0.083
Comp. SF. Cichorioideae	0.051	0.244	0.065	*Ranunculus acris*-t	0.014	3.848	0.718
Corylus	0.025	1.400	0.042	Rubiaceae	0.019	3.946	0.589
Cyperaceae	0.035	1.002	0.164	*Rumex acetosa-t**	0.018	1.559*	0.089*
Fagus	0.057	6.667	0.173	*Salix*	0.022	1.267	0.313
Filipendula	0.006	2.480	0.821	*Secale*-t	0.060	3.017	0.052
Fraxinus	0.022	0.667	0.027	*Tilia*	0.032	0.800	0.029
Juniperus	0.016	2.067	0.036	*Ulmus*	0.032	1.267	0.050

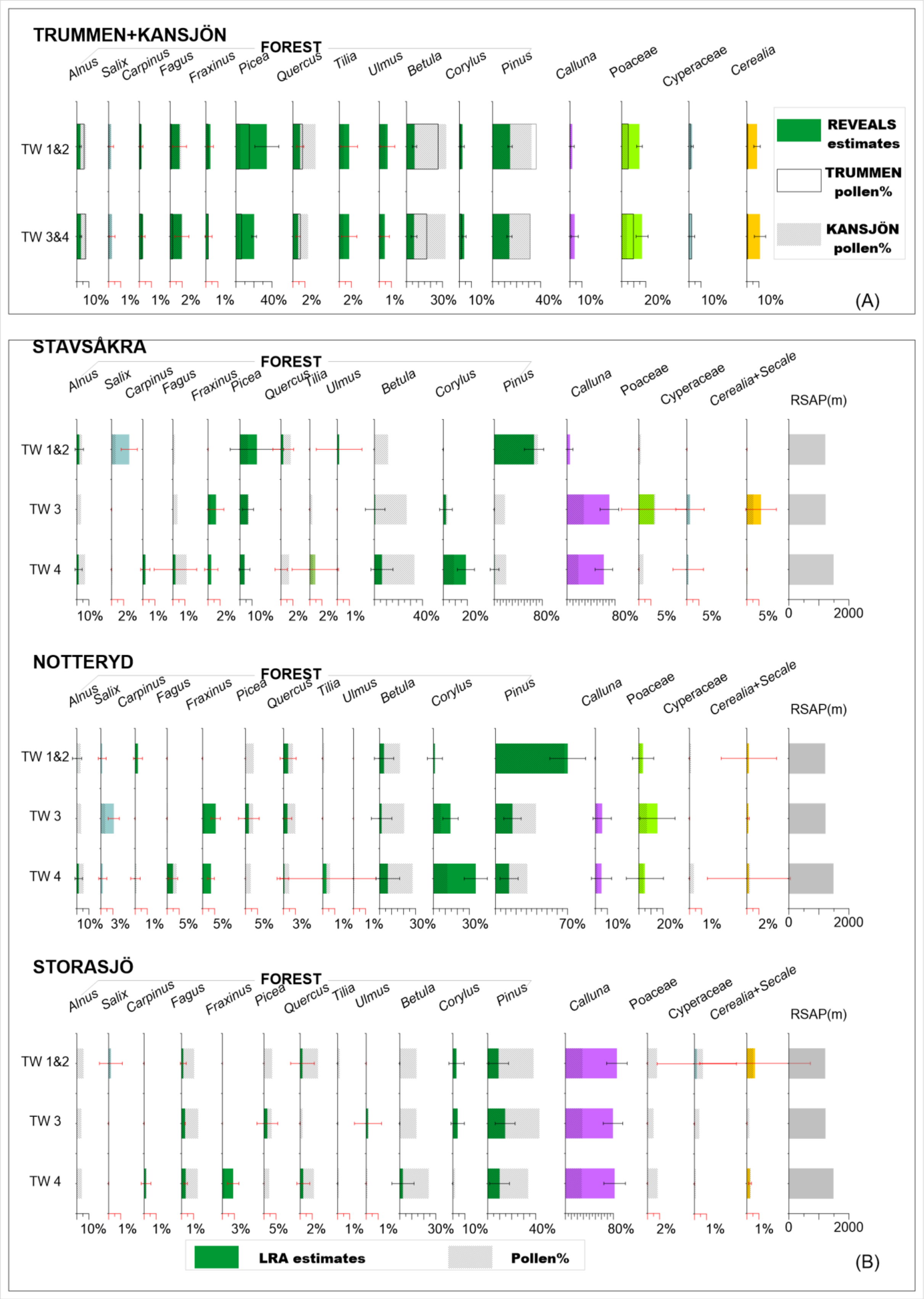

Figure 3 Taxon-based REVEALS estimates of regional plant abundance using the pollen records Trummen and Kansjön (two large lakes) and taxon-based LRA estimates of local

weighted plant abundance (LRA-DWtaxon) using the pollen records from Stavsåkra, Notteryd and Storasjö (two small bogs) within the relevant source area of pollen (RSAP *sensu* Sugita, 1994) for four time windows: AD 1950-2010 (TW1), AD 1925-1950 (TW2), AD 1825-1925(TW3) and AD 1685-1825 (TW4).

References

Cui, Q.-Y. (2013) Fire history in the hemiboreal and southern boreal zones of southern Sweden during 11 000 years. Linnaeus University Dissertations no 155/2013. Linnaeus University Press, ISBN: 978-91-87427-62-6, pp. 45.

Cui, Q.-Y., Gaillard, M.-J., Lemdahl, G., Sugita, S., Greisman, A., G., J. & Olsson, F. (2013) The role of tree composition in Holocene fire history of the hemiboreal and southern boreal zones of southern Sweden, as revealed by the application of the Landscape Reconstruction Algorithm — implications for biodiversity and climate-change issues. *The Holocene*, doi: 10.1177/0959683613505339, 1-17.

Berglund, B.E. (1991) The cultural landscape during 6000 years in southern Sweden: the Ystad project. *Ecological Bulletins* 41: 1–495.

Emanuelsson, U. (2007) *The rural landscapes of Europe – How man has shaped European nature.* Formas, Stockholm.

Gaillard-Lemdahl, M., Birks, H.J.B., Emanuelsson, U., Berglund, B.E. (1992). Modern pollen/land-use relationships as an aid in the reconstruction of past land-uses and cultural landscapes: an example from South Sweden. Vegetation History and Archaeobotany. 1. 3-18.

Gaillard-Lemdahl, M., Birks, H.J.B., Emanuelsson, E., *et al.* (1994). Application of modern pollen/land-use relationships to the interpretation of pollen diagrams - reconstructions

of land-use history in South Sweden 3000-0 BP. *Review of Palaeobotany and Palynology*. 82. 47-73.

Gaillard, M.J., Sugita, S., Mazier, F., *et al.* (2010) Holocene land-cover reconstructions for studies on land cover-climate feedbacks. *Climate of the Past*, **6**, 483-499.

Gregory, P. H. (1973) *The microbiology of the atmosphere*. Leonard Hill, Aylesbury.

Greisman, A. (2009) *The Role of Fire and Human Impact in Holocene Forest and Landscape Dynamics of the Boreo-Nemoral Zone of Southern Sweden – A Multiproxy Study of Two Sites in the Province of Småland*. Dissertation Series 62. Kalmar: Faculty of Natural Sciences, University of Kalmar.

Greisman, A. and Gaillard, M.-J. (2009) The role of climate variability and fire in early and mid-Holocene forest dynamics of southern Sweden. *Journal of Quaternary Science*, **24**, 593–611.

Hellman, S., Gaillard, M.-J., Broström, A. et al. (2008a) Effects of the sampling design and selection of parameter values on pollen-based quantitative reconstruction of regional vegetation: A case study in southern Sweden using the REVEALS model. *Vegetation History and Archaeobotany,* **17**, 445–459.

Hellman, S., Gaillard, M.-J., Broström, A. et al. (2008b) The REVEALS model, a new tool to estimate past regional plant abundance from pollen data in large lakes: Validation in southern Sweden. *Journal of Quaternary Science,* **23**, 21–42.

Mazier, F., Gaillard, M.J., Kunes, P., Sugita, S., Trondman, A.K. & Brostrom, A. (2012) Testing the effect of site selection and parameter setting on REVEALS-model estimates of plant abundance using the Czech Quaternary Palynological Database. *Review of Palaeobotany and Palynology*, **187**, 38-49.

Mazier, F., Broström, A., Bragée, P., Fredh, D., Stenberg, L., Thiere, G., Sugita, S. & Hammarlund, D. (in review). Two hundred years of changing land-use in the southern Swedish Uplands: pollen-based reconstructions using the Landscape Reconstruction Algorithm compared with historical maps. *Review of Palaeobotany and Palynology*.

Nielsen, A.B., Giesecke, T., Theuerkauf, M., Feeser, I., Behre, K.E., Beug, H.J., Chen, S.H., Christiansen, J., Dorfler, W., Endtmann, E., Jahns, S., de Klerk, P., Kuhl, N., Latalowa, M., Odgaard, B.V., Rasmussen, P., Stockholm, J.R., Voigt, R., Wiethold, J. & Wolters, S. (2012) Quantitative reconstructions of changes in regional openness in north-central Europe reveal new insights into old questions. *Quaternary Science Reviews*, **47**, 131-149.

Nielsen, A.B. & Sugita, S. (2005) Estimating relevant source area of pollen for small Danish lakes around AD 1800. *Holocene*, **15**, 1006-1020.

Olsson, F., Gaillard, M.J., Lemdahl, G., Greisman, A., Lanos, P., Marguerie, D., Marcoux, N., Skoglund, P. & Waglind, J. (2010) A continuous record of fire covering the last 10,500 calendar years from southern - Sweden The role of climate and human activities. *Palaeogeography Palaeoclimatology Palaeoecology*, **291**, 128-141.

Prentice, I.C. (1985) Pollen representation, source area, and basin size – toward a unified theory of pollen analysis. *Quaternary Research*, **23**, 76-86.

Stuart, A. & Ord, J.K., 1994. Kendall's Advanced Theory of Statistics. Volume 1. Distribution Theory. Edward Arnold, London.

Sugita, S. (1994) Pollen representation of vegetation in Quaternary sediments – Theory and method in patchy begetation. *Journal of Ecology*, **82**, 881-897.

Sugita, S. (2007b) Theory of quantitative reconstruction of vegetation I: pollen from large sites REVEALS regional vegetation composition. *Holocene*, **17**, 229-241.

Sugita, S., Parshall, T., Calcote, R. & Walker, K. (2010) Testing the Landscape Reconstruction Algorithm for spatially explicit reconstruction of vegetation in northern Michigan and Wisconsin. *Quaternary Research*, **74**, 289-300.

Örback, A. I. E. (1998) Lantmäterikartorna som historisk källa. *Sveriges Nationalatlas, Sveriges kartor* (ed. by U. Sporrong and H.-F. Wennström), pp. 22-25. Lantmäteriverket, Stockholm.
